# Supplementary material for: Inflammatory factors and risk of lung adenocarcinoma: a Mendelian randomization study mediated by blood metabolites
Source: Front Endocrinol (Lausanne). 2024 Aug 27;15:1446863. doi: 10.3389/fendo.2024.1446863 (PMC11384989; doi:10.3389/fendo.2024.1446863)
Supplement: Supplementary file 1 [file DataSheet1.zip › supplementary files/S2/GCST90200335/forest.pdf]

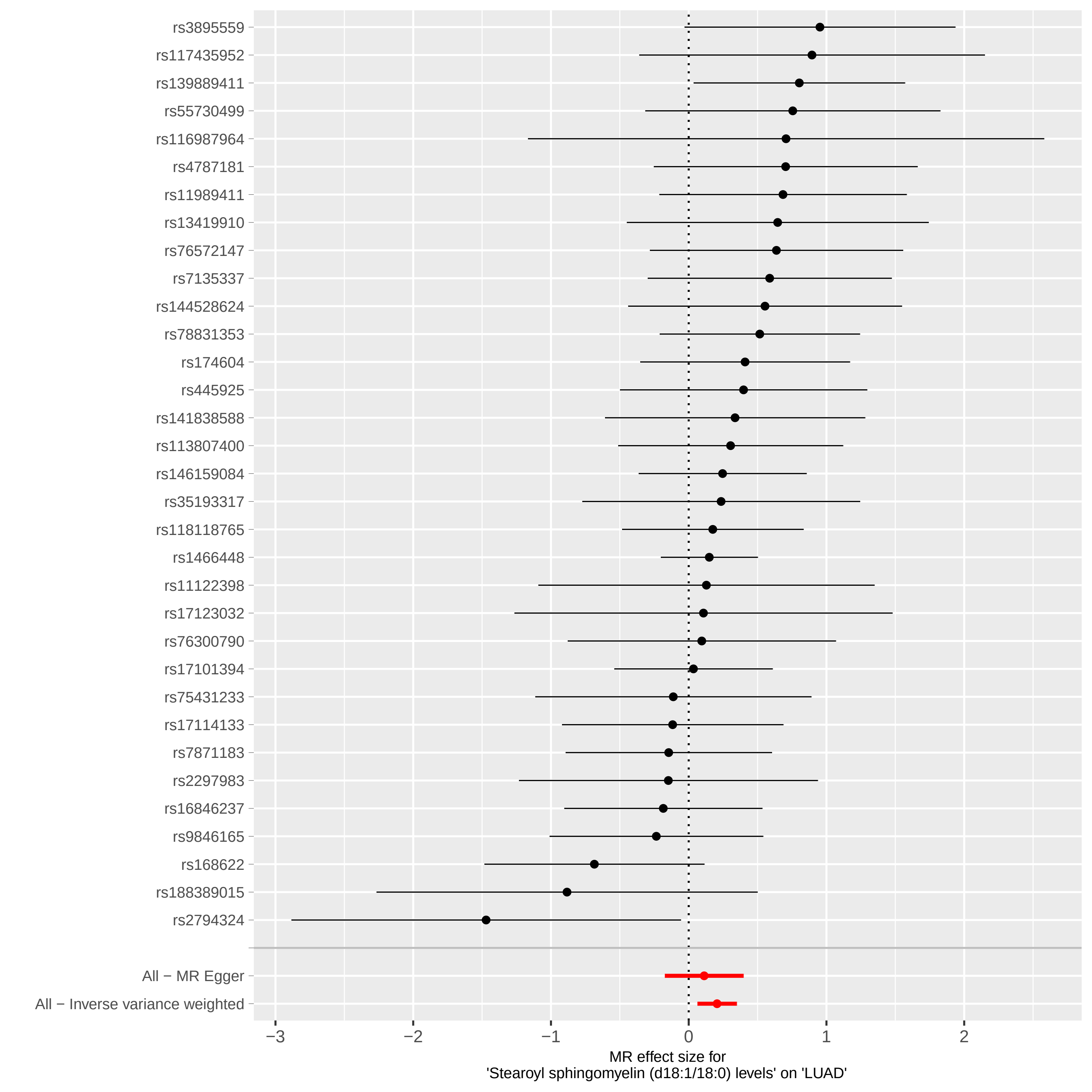
rs3895559
rs117435952
rs139889411
rs55730499
rs116987964
rs4787181
rs11989411
rs13419910
rs76572147
rs7135337
rs144528624
rs78831353
rs174604
rs445925
rs141838588
rs113807400
rs146159084
rs35193317
rs118118765
rs1466448
rs11122398
rs17123032
rs76300790
rs17101394
rs75431233
rs17114133
rs7871183
rs2297983
rs16846237
rs9846165
rs168622
rs188389015
rs2794324
All − MR Egger
All − Inverse variance weighted
−3
−2
−1
0
1
2
MR effect size for
'Stearoyl sphingomyelin (d18:1/18:0) levels' on 'LUAD'
